# The effects of the transition from home-based childcare to childcare centers on children’s health and development in Colombia

**DOI:** 10.1016/j.ecresq.2018.08.005

**Published:** 2019

**Authors:** Raquel Bernal, Orazio Attanasio, Ximena Peña, Marcos Vera-Hernández

**Affiliations:** a Universidad de los Andes, Department of Economics, Centro de Estudios sobre Desarrollo Económico, Calle 19A # 1-37E, Edificio W, Bogotá, Colombia; b University College London, Department of Economics, Gower Street, London WC1E6BT, UK

**Keywords:** Colombia, Child development, Home-based childcare, Center-based childcare, Poverty, Nutrition

## Abstract

Colombia’s national early childhood strategy launched in 2011 aimed at improving the quality of childcare services offered to socio-economically vulnerable children, and included the possibility that children and their childcare providers could transfer from non-parental family daycare units to large childcare centers in urban areas. This study seeks to understand whether the offer to transfer and the actual transfer from one program to the other had an impact on child cognitive and socioemotional development, and nutrition, using a cluster-randomized control trial with a sample of 2767 children between the ages of 6 and 60 months located in 14 cities in Colombia. The results indicate a negative effect of this initiative on cognitive development, a positive effect on nutrition, and no statistically significant effect of the intervention on socioemotional development. We also explored the extent to which these impacts might be explained by differences in the quality of both services during the transition, and report that quality indicators are low in both programs but are significantly worse in centers compared to community nurseries.

## Highlights

• We estimated the effects of the offer to transfer from home-based childcare to childcare centers in Colombia.
• The evaluation design is based on a cluster-randomized control trial in 14 cities.
• We report positive effects on children’s nutritional status and negative effects on cognitive development.
• We find that the offer to transfer increased the probability of center attendance and decreased the probability of home-based childcare enrollment.
• We report that indicators of infrastructure quality are better in centers than in home-based nurseries but the opposite occurs with indicators of the quality of routines and activities.

## 1 Introduction

An overwhelming 250 million children younger than 5 years of age in developing countries have been identified as being at risk of not reaching their full developmental potential because they live in poverty and face the psychosocial and material challenges associated with economic adversity (Black et al., 2017). These children have higher probabilities of underperforming at school and, thus, of reaching adulthood with lower earnings capacity, higher probability of risky behaviors and lower overall quality of life (Britto et al., 2017).

The period between birth and 5 years of age has been identified as critical to alter the long-term developmental trajectories of individuals (Cunha, Heckman, Lochner, & Masterov, 2006; Engle et al., 2007; Heckman, 2006; Yoshikawa et al., 2013). Although there is increasing agreement about the value of early interventions, there is limited consensus about the specific types of interventions that are most effective. Most of the available evidence is based on a few rigorous small randomized trials for developed countries while developing nations usually have limited resources and capacity to conduct longitudinal or randomized control trial evaluations of large-scale programs (Britto, Yoshikawa, & Boller, 2011).

While the initial focus in low- and middle-income countries (LMIC) was on increasing access to early childhood education (ECE), the emphasis has now shifted to quality, particularly where enrollment rates are high (Yoshikawa et al., 2015). However, there is little evidence on how to increase quality effectively at scale (Britto et al., 2017). Several LMIC initially expanded early education services through low-cost home-based childcare (Nores & Barnett, 2010). Often these home-based interventions are contrasted with considerably more expensive center-based ones, under the presumption that the latter are of higher quality than the former. This study provides a direct and rigorous comparison between these two types of childcare programs. In particular, we take advantage of the national roll-out of a massive transition from home-based programs to center-based child care in Colombia to assess the effects of the offer to be part of such transition on child development.

## 2 Background and early childhood policy in Colombia

In Colombia, 2.8 million children younger than 6 years of age live in poverty, 14% of these poor children are stunted and their scores in receptive language are one standard deviation below those of their peers in higher SES households (Bernal & Quintero, 2014). Among socio-economically vulnerable children 0–6 years old, enrollment in public ECE programs ranged from 20% to 40% for most of the period since the late 1980s (Bernal & Camacho, 2011). All public ECE programs are targeted to children and families in poverty (representing close to 65% of the total population). More than half of the provision was through small home-based community nurseries (*Hogares Comunitarios de Bienestar* or HCBs henceforth) since the 1980s. HCBs are non-parental family childcare units where care is provided in the childcare provider’s own home. HCBs are run by one woman in the community known as “community mother” (MC). Each HCB serves 12–15 children between ages 6 months and 6 years from poor households in full-day schedules, and provide 70% of daily nutritional requirements. MCs typically have a high school degree (67.3%) or some higher education (17%), with a smaller percentage having only elementary education (15%). MCs are required to attend a 40 h pre-service training. The average cost of the program is 440 US dollars (USD) per child per year.

Recent evaluations of the HCB program have found positive impacts on children’s height (Attanasio, Di Maro, & Vera-Hernández, 2013), as well as positive effects on cognitive and socio-emotional development associated with long exposure to the program (Bernal & Fernández, 2013); however, these evaluations also reported severe deficiencies in the quality of care provided. Both papers found that extended exposure to the program significantly improved the condition of very disadvantaged children, despite quality issues, probably because of very poor learning environments at home (the counterfactual).

Around 2011, nearly 1.2 million children were being served by community-based programs (enrollment of about 25–30% of eligible populations), mainly HCBs, while only about 130 thousand children attended public center-based care. For that reason, very little was known about the quality and impacts of center-based childcare at the time. Apart from these public services provided through the social protection system, specifically the Colombian Family Welfare Agency (*Instituto Colombiano de Bienestar Familiar* or ICBF henceforth), there was also a supply of private preschools targeting socioeconomically vulnerable populations. In 2010, close to 60% of all poor children attending some ECE program attended public programs provided by ICBF or local governments, while the other 40% was provided privately. Between 2010 and 2013 some public schools in the formal education sector, especially in big cities, introduced one preschool grade. The latter accounted for 6% of total ECE enrollment of low SES populations in 2013.

In 2011, the government launched the national early childhood strategy *De Cero a Siempre* (From Zero to Forever, DCAS) aimed at increasing access and, most importantly, improving the quality of early childhood services provided to poor children. The objective was to deliver high-quality and comprehensive early childhood services for 1.2 million disadvantaged children under the age of 6 with a budget close to USD 1,290 million dollars per year over 4 years (Bernal and Camacho, 2014).

The main hypothesis of the DCAS theory of change is that given the holistic nature of early childhood development, it is critical to provide fully integrated/comprehensive early childhood services to successfully promote early development (*Comisión Inter-Sectorial para la Primera Infancia* [CIPI], 2013). The Inter-sectorial Board for Early Childhood (CIPI) defined a service or program as fully integrated if it could provide concurrently: childcare, health and nutrition, early education, recreation and the exercise of the child’s rights as a citizen. In light of this approach, HCBs were not deemed as fully integrated services by the time DCAS was introduced. For this reason, one of the initial pillars of the DCAS strategy was to offer children the possibility to transfer from HCBs to large childcare centers (*Centros de Desarrollo Infantil* or CDIs henceforth) in urban areas. Center-based care has grown from serving about 125,000 children up to 2011 to about 380,000 children in 2016. The strategy was based on the assumption that CDIs could provide fully integrated services, which we discuss in detail below.

In this study, we investigated whether the offer to transfer from HCBs to CDIs had impacts on child cognitive and socioemotional development, and nutrition. We also sought to understand some mediational pathways that could potentially explain these effects. In particular, we studied the differences in quality between the two programs and discuss how these could have mediated the estimated impacts. With this objective, in 2010, we designed and implemented a clustered-randomized trial to evaluate the impacts of the offer to transfer from HCBs to CDIs in Colombia.

## 3 The intervention

CDIs are different from HCBs in a variety of ways. First, each CDI serves close to 300 children between the ages of 6 months and 5 years of age in buildings specifically designed for early education (compared with 12–15 children being cared for in the provider’s own household in HCBs) with specialized areas such as playground and refectory. An appropriate infrastructure is often thought of as a minimum requirement for quality in terms of suitable physical spaces and basic safety features. CDI slots were first offered to children who were in nearby HCBs. If these children did not fill all available vacancies, the remainder slots were offered to eligible children residing in the community. MCs with at least a high-school degree could transfer to CDIs as classroom teachers.

Second, CDIs are organized in same-age classrooms, while HCBs serve children from 6 months to 5 years of age in the same physical space. There is mixed evidence regarding the benefits of age-specific preschool classrooms with respect to mixed-age classrooms. Moller, Forbes-Jones, and Hightower (2008) argued that same-age classrooms allow teachers to target their curriculum more specifically for a particular age range, which allows them to focus on what is developmentally appropriate for that group. On the other hand, Guo, Tompkings, Justice, and Petscher (2014) found positive effects of classroom variation in age composition on children’s vocabulary gains for younger but not for older children. Similarly, Bandura (1986) showed that in age-mixed classrooms, young children learn by emulating and observing older children, and Derscheid (1997) provided evidence that older children practice prosocial behaviors by supporting and mentoring younger children. However, Goldman and Chaillέ (1984) found no evidence that in mixed-age classrooms, older children engage in nurturing and mentoring relationships with younger children that might enhance child development as compared to same-age groups.

Third, in addition to teachers, CDIs also hire housekeeping and administrative staff (e.g., kitchen, cleaning, and center director) as well as a nutritionist and a psychologist, allowing teachers to devote more time to classroom activities as compared to MCs in HCBs who have to cook, clean and run their own service unit, in addition to caring for the children. At the same time, teachers have to comply with operational guidelines (administrative, not pedagogical) regarding preparation of materials, documentation and child assessments, reports to parents, safety in the classroom, etc. These procedures are not as structured and certainly not well monitored in HCBs. Andrew et al. (2016), who assessed the effects of the presence of the interdisciplinary group of professionals in Colombian childcare centers on children’s development, reported that only about 38% of centers fully complied with these staffing guidelines. The main tasks of the nutritionists include administration of all processes related to food provided to children in centers, revision of menus, supervision of portions served, monitoring of special cases, anthropometric follow-up, training kitchen personnel and working with families on better nutrition and health-related habits. On the other hand, psychologists' main tasks are to support parents on all issues within the socio-emotional domain, handle domestic violence cases, diagnose and prepare plans for cases requiring follow-up, in particular, those in which developmental lags have been identified (Andrew et al., 2016).

Fourth, the child-teacher ratio is worse in CDIs than in HCBs (25:1 in CDI and 12:1 in HCB). The literature reveals that programs with class sizes of at most 20 children can be shown to produce large gains for disadvantaged children (Barnett & Boocock, 1998). More generally, a large body of literature points to the links between staff–child ratios, program quality, and child development. In particular, it has been shown that with a ratio of about 10 children per adult, children have greater opportunities for learning (Bowman, Donovan, & Burns, 2001).

Fifth, both CDIs and HCBs provide approximately 70% of daily nutritional needs. In both cases, a morning and afternoon snack is provided, as well as lunch. However, servings might be different in CDIs with respect to HCBs due to the presence of a professional nutritionist in CDIs.

There is no specific curricular guideline, that is, neither in CDIs nor in HCBs. The CIPI has emphasized that teachers and service providers must have the freedom to choose their own curricular guidelines and contents. For this reason, there is no national curriculum, and standards are intentionally broad. That means that both, teachers in CDIs and MCs in HCBs, are expected to adapt the learning standards to their own classrooms and contexts (CIPI, 2013). Teacher training and coaching strategies are not common in ECE programs in Colombia and vary significantly across providers for both CDIs and HCBs. Yoshikawa, Weiland, and Brooks-Gunn (2016) presented recent evidence about the positive effects of the use of a structured preschool curriculum focused on a particular set of skills incorporating engaging, play-based activities on childcare quality and child development. Furthermore, the authors emphasized that a developmentally focused curricula, combined with intensive in-service training or coaching for teachers, has been shown to improve the quality of preschool instruction.

It is important to note that all public ECE services (including CDIs and HCBs) have comprehensive operational and technical guidelines (referents) that relate mostly to administrative guidelines and certain structural service parameters such as the number of children per square meter, characteristics of physical areas, teachers’ qualifications, food handling, bookkeeping, etc., but do not provide specific pedagogical directives.

The annual cost per child in a CDI is much higher than in a HCB, at 1600 USD (excluding the cost of the infrastructure). Both programs are run by the ICBF. DCAS has enrolled approximately 235 thousand children younger than 6 years in CDIs since 2011, out of which at least half were children who were transfered out of HCBs into CDIs.

The theory of change behind the introduction of CDIs indicates that, on one hand, age-specific grouping of children, better infrastructure, more time available for teachers to spend in classroom activities and staffing of centers with professionals could improve teaching practices and routines in the classroom, as well as improve parental practices at home through a more close relationship with the psychologist and nutritionist. If this were the case, the changes could in turn, improve cognitive and socio-emotional development of children. One could also expect the presence of a nutritionist and specialized kitchen personnel in centers to be related with improved food handling and content of food servings, improved nutritional habits, and better monitoring of children’s nutritional status, and thus, improvements in children’s nutrition and health.

On the other hand, while more time is given to teachers in CDIs to spend with children (vs. other tasks such as food preparation), it is also possible that children in HCBs benefit from the smaller adult-to-child ratio. In addition, CDI teachers might feel less accountable to parents than MCs because CDI teachers are part of a larger team, and might care for different children every year. In sum, there are both positive and negative potential effects of transitioning from HCBs to CDISs and hence, the overall magnitude and direction of the impact cannot be clearly determined *ex ante*.

## 4 On the quality of early education services

In LMICs there have been few comprehensive educational interventions as the expansion of early childhood services occurred through less costly alternatives such as home-based childcare or conditional cash transfer programs (Nores & Barnett, 2010). As a result, there is very little empirical evidence about the effects of more comprehensive, potentially higher quality, but also more expensive early education programs. This study contributes to the understanding of the effects of center-based care as result of a specific offer to transfer out of home-based services and into childcare centers.

The evidence resulting from developed countries, especially the United States, based on small randomized control trials of intensive, high-quality early educational interventions finds positive effects on child development and suggests that the extent to which children benefit from center-based childcare can vary depending on its quality (Britto et al., 2011; NICHD, 2000; Cleveland & Krashinsky, 2013). Quality has different dimensions, including structural and process quality. Structural quality refers to features such as class size, child–adult ratio, teacher qualifications, and physical environment, while process quality is associated with features such as teacher–child interaction and the environment in which children learn (Yoshikawa et al., 2015).

There seems to be an agreement that measures of process quality are critical aspects that promote child development (NICHD, 2000; Yoshikawa et al., 2016). The evidence from developed countries suggests that childcare of good quality has positive strong effects on children’s development and health that are longlasting and sizable relative to its cost (Cunha et al., 2006; Camilli, Vargas-Baron, Ryan, & Barnett, 2010). There is still little evidence on center-based childcare programs on a large scale. Engle et al. (2011) reviewed research on center-based early childcare in LMICs and concluded that most effects on children’s cognitive functioning, school readiness, and school performance were observed in cases of high-quality programs. Similarly, Britto et al. (2017) reviewed the evidence on the effects of early education programs in developing countries, and concluded that regardless of the type of program, quality is a key predictor of its effectiveness, in particular, factors such as positive interactions, individualized attention and positive emotional climate. This study contributes to this literature by comparing the impacts of two different programs on a large scale, which are different in terms of components of ECE quality.

As can be inferred from the earlier description of the intervention, most differences between CDIs and HCBs are related to features of structural quality: adult–child ratios, group sizes and their age composition, infrastructure and basic safety, and qualified personnel. Features of process quality such as a developmentally focused curriculum, teacher pedagogical and classroom organization skills, and warm and responsive environments (Yoshikawa et al., 2016) are not explicitly part of the transfer from HCB to CDI. Thus, we measured process quality at endline and our statistical analysis allows for the possibility of positive or negative treatment effects on child development.

## 5 Methods

### 5.1 Study design

We evaluate the impact of the offer to transfer from HCBs to CDIs using a cluster-randomized effectiveness trial in 14 cities in Colombia involving 15 CDIs. These cities include the entire pipeline of centers under construction at the time that the evaluation started. The initiative to build a center came from each of the municipalities, as the infrastructure was funded directly by the local government, while operating expenses of centers are funded at the national level. The cities are medium to large, with reasonable institutional and financial capacity. In particular, seven of the 14 cities had between half a million a 1.2 million inhabitants at the time of the study, and the other seven cities had between 112 thousand and 270 thousand inhabitants.

The study took place between November 2010 and November 2012. For each CDI in the sample, we identified 28 HCBs located in the vicinity. Up to 5 HCBs of these were directly offered to transfer to the CDI because of extreme poverty conditions or close proximity. From the remaining HCBs, we randomly selected 20 to make part of the study. We then randomly assigned 15 of these to *receive an offer* to transfer to a CDI while the remaining five did not receive this offer. The former constitutes our treatment group (intent-to-treat group), while the latter are our control group.

The ICBF informed MCs in HCB units randomly assigned to the treatment that they had been selected − and were strongly encouraged − to transfer to the closest CDI along with the children they served at the time. However, the ICBF did not force the transfer if a MC refused the offer.^2^ Parents in treated HCBs were supposed to be informed about and encouraged by their MC to transfer their children with her, but were not directly contacted by ICBF officers or the research team. Even if informed by the MC, parents were not obliged to transfer to a CDI if they did not want to, and could look for other HCBs or alternatives sources of childcare.

MCs assigned to the control group were never contacted by the ICBF in regards to a possible transition to the CDIs. Regardless of the random assignment status of an HCB, and whether or not MCs assigned to treatment had encouraged parents to transfer to the nearest CDI, it is possible that parents (and MCs) in HCBs in both study groups would know about the construction of CDIs and could have inquired about available slots. CDIs did not deny available slots to children if they fulfilled the age and poverty eligibility criteria.

Given these issues, the design of the evaluation is not based on the random assignment of the transfer from an HC to a CDI but is based on the randomized encouragement or promotion to a transition. Children attending ‘treated’ HC at baseline do not necessarily enroll into CDI and children attending ‘control’ HC might transfer to CDIs. We discuss the implications of these issues for the evaluation below.

Each cluster (one HCB) had an average of 9.2 children aged 6-60 months at baseline. We excluded children older than 60 months at baseline because they would have been served by the intervention for a very short period before leaving for elementary school. Baseline data collection occurred between November 2010 and May 2011. Follow-up data collection took place in two different stages to accommodate the budgetary constraints of the study funder. The first stage took place in November and December 2011 in six out of 15 CDIs in the sample. The second stage took place from September to November 2012 in the remaining nine CDIs. Different opening dates of CDIs and different timings of follow-up data collection implied three different groups of CDIs in terms of how long children could have been exposed to the treatment. In a first group of six CDIs, nine to ten months elapsed between the opening of centers and follow-up data collection. In a second group of six CDIs, this period was closer to 19 months. Finally, in a third group of three CDIs six to eight months elapsed. Differences in center opening dates occurred due to delays in their construction associated with *“La Niña”* rain season in 2011.

### 5.2 Study sample

**Fig. 1:**
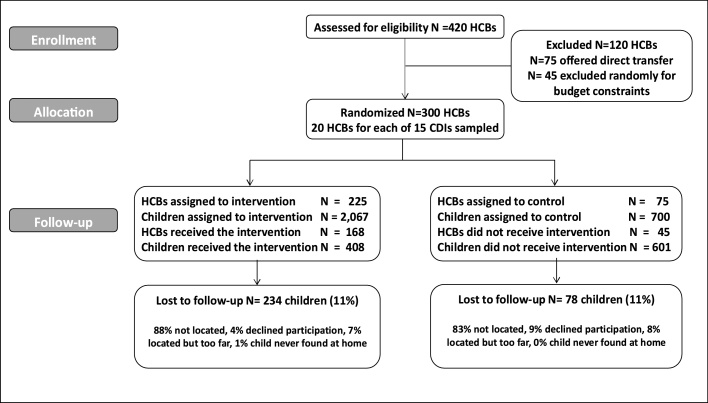
Study design.

Source: Consort flow chart. Own elaboration.

This protocol implied a total sample of *N* = 2767 children younger than 60 months of age and 300 HCBs assessed at baseline. To determine the sample size for the trial, we assumed an average intervention effect of 0.20 SD of a standardized score, based on Nores and Barnett (2010) who reported effect sizes of about 0.25 SD for continuous outcomes in cognition for early education programs in the region. We estimated the conditional intracluster correlation to be 0.0135, using height *Z*-scores based on data from Bernal and Fernández (2013). Finally, we assumed a 10% attrition rate between baseline and follow-up. Power calculations yielded a 90% power (with *∝* = 0.05) for a sample of nine children per HCB for a total of 300 HCBs in an array of 75% treated and 25% controls. In sum, 225 HCBs (2067 children) were assigned to receive an offer to transfer to CDIs, and 75 HCBs (700 children) did not receive the offer to transfer (see Fig. 1).

At follow-up, 2455 children and their families were re-interviewed (89%). Using a linear probability model for the likelihood of attrition, we found that random assignment to treatment (*ITT*) does not predict attrition, but household poverty and parental absence do. We controlled for both in the econometric analyses. We also observed that treated and control attritors were not different at baseline except for household poverty with the former being poorer than the latter.

### 5.3 Data collection procedures

Informed consent was obtained from all subjects in this study. Ethics Committees at participating institutions approved the study’s protocol in 2010. Parent questionnaires on household’s socio-demographic characteristics, children’s socio-emotional outcomes and cognitive and motor outcomes (by the Ages & Stages questionnaire) were collected by parental report at HCBs at baseline and HCBs/CDIs at follow-up. In case children did no longer attend early education services at follow-up these were collected at the child’s household. Data on child’s enrollment status at follow-up were collected from administrative records and parental report. Children were assessed by Woodcock-Muñoz III in HCBs or CDIs and mothers were invited to be present. Tester held degrees in psychology and had four weeks’ training, including practice sessions with children of the target age groups. The inter-rater reliability (intracluster correlation) was above 0.9 on each subscale of the Woodcock-Muñoz III.^3^ When it was not possible to assess children in service units, they were assessed at their own homes trying to guarantee similar testing conditions. Classroom quality measures by ECERS-R, ITERS-R and FDCRS (details below) were completed by psychologists who observed CDI classrooms and HCBs for at least half of a school day. Assessors were trained to be unobtrusive, to the extent possible, in the classroom or HCB while observing and recording. The team of six psychologists were trained for three weeks and assessed for reliability in two centers and 10 HCBs in Bogotá, which were not in the study sample. The inter-reliability was above 0.8 on all subscales of the three instruments.

### 5.4 Measures

#### 5.4.1 Household demographic and socio-economic status

Household demographic and socio-economic status data were collected at baseline and follow-up. The survey included information on household characteristics: basic utilities and characteristics of the home; income; durable goods ownership; parental education; presence of the father; employment status; marital status; household size and composition, and parental practices. Using principal-component analysis, we constructed a wealth index on a subset of household characteristics. We then used this index to classify households into wealth quintiles.

#### 5.4.2 Nutrition

We collected information on height, weight and arm circumference at baseline and follow-up following World Health Organization (WHO) standards. Based on these measures, we constructed several nutritional indicators (WHO Multicentre Growth Reference Study Group, 2006/7). No reference for mid-upper-arm circumference for 5–19 year olds was included in the WHO, 2007 revision. For this reason, this variable was used only for a subsample of children younger than five at follow-up or not used at all.

#### 5.4.3 Health

We measured health by: (1) blood haemoglobin by a finger prick assay with HemoCue Hb 201+ devices for all children at follow-up and we construct the fraction of anemic children as the percentage of children under age 5 with haemoglobin levels below 100 g/l and below 110 g/l for children older than 5; and (2) parasitic examination in stool sample, for a random subsample of 75% of all children at follow-up only due to budgetary constraints.

#### 5.4.4 Cognitive development

(1) We used the Ages & Stages Questionnaire (ASQ), third edition, for all children at baseline and follow-up (Squires & Bricker, 2009). The ASQ is a questionnaire focusing on cognitive development and the identification of children at risk of cognitive developmental difficulties. It includes fine and gross motor, communication, and problem solving. The ASQ has been used for early development assessments in LMICs (Rubio-Codina, Araujo, Attanasio, Muñoz, & Grantham-McGregor, 2015). The Cronbach’s *α* is 0.87 for total ASQ scores (by sbuscale, the Cronbach’s *α* was 0.61 for communication, 0.67 for problem resolution, 0.76 for gross motor and 0.70 for fine motor). We report and use nonparametrically age-standardized scores. It is worth noting that the ASQ is generally regarded as a screener test and not considered a gold-standard measure of child development. For this reason, we are careful in the interpretation of these results. (2) The Woodcock-Muñoz III Tests of Achievement (WM-III) for a random subsample of 75% children older than 30 months of age at follow-up. The WM-III is a set of individually administered tests of children’s early literacy and mathematical skills and knowledge, and we use subtests 1, 5, 6, 7, 10, and 14, to measure general verbal ability and receptive language, associative memory, attention, and mathematical reasoning (Muñoz-Sandoval, Woodock, McGrew, Mather, & Schrank, 2005). The WM-III has been used to evaluate effects of early childhood interventions in Latin-American contexts (Fernald, Kariger, Engle, & Raikes, 2009). We report age-standardized scores using the Compuscore software. We computed composites directly in the field and do not have individual item scores available. However, we found that all subscales positively and significantly correlate with maternal education and household wealth.

#### 5.4.5 Socio-emotional development

We used the ASQ for the socio-emotional domain (ASQ:SE) (Squires, Bricker, & Twombly, 2009) for all children at baseline and follow-up. The ASQ:SE focuses on socio-emotional development and the identification of children at risk of social-emotional difficulties. It includes self-regulation, compliance, communication, adaptive functioning, autonomy, affect, and interaction. The ASQ:SE has been used for early development assessments in LMICs (Handal, Lozoff, Breilh, & Harlow, 2007; Heo, Squires, & Yovanoff, 2007). The Cronbach’s α for total ASQ:SE scores is 0.70 but there is significant variation by subscale (self-regulation = 0.50, compliance = 0.49, communication = 0.34, adaptive function = 0.21, autonomy = 0.13, affect = 0.25 and interaction = 0.36). We report raw scores in the descriptive statistics and use nonparametrically age-standardized scores in the statistical analysis. Again, we note that ASQ:SE is generally regarded as a screener test and not considered a gold-standard measure of child development.

#### 5.4.6 Class environment and activities

We used three of the Environment Rating Scales (ERS) to measure classroom quality for the different childcare programs assessed in this study. The ERS scales provide a global measure of preschool classroom quality with complimentary scales assessing different segments of the childcare spectrum. In particular, the Early Childhood Environmental Rating Scale − Revised (ECERS-R; Harms, Clifford, & Cryer, 1998) is designed for preschool aged children in center-based care; the Infants and Toddlers Environmental Rating Scale − Revised (ITERS-R; Harms, Cryer, & Clifford, 2003) for infants and toddlers in center based care; and the Family Daycare Rating Scale (FDCRS; Harms & Clifford, 1989) provides a global measure of environmental quality for children in home-based childcare settings such as HCBs. The instruments cover a broad range of quality considerations from safety to teacher–child interaction to parent involvement based on checklists that range from 39 to 43 items.

These measures have been used extensively in the field and have well-established validity and reliability. ERS scales have been used in a wide range of countries with different cultures and economic contexts. The ERS scales have shown predictive validity to child gains across cognitive domains (Burchinal et al., 2000) and social–emotional domains (Sylva et al., 2006).

ECERS-R, ITERS-R and FDCRS were completed by psychologists who observed CDI classrooms and HCBs for at least half of a school day. Each assessor rates each of 43 items in ECERS, 39 in ITERS and 40 in FDCRS. All items are presented as a 7-point scale with descriptions of what is required under four quality levels: 1 (inadequate), 3 (minimal), 5 (good) and 7 (excellent). Each description consists of one or more numbered indicators that must be scored by observation and evaluation of a classroom. The total number of items in each scale is organized into seven subscales that provide a practical and conceptual organization for the items. The following are the seven elements of preschool quality considered by ERS subscales: physical environment; basic care; pedagogical activities and curriculum; interaction; schedule and program structure; and parent and staff education. Most of the items examine the quality of what children actually experience in the program. A small subset of items looks into the quality of provisions for the adults involved in the program, i.e., parents and staff.

In sum, the ERS scales include elements related to structural quality such as the physical environment, as well as elements related to process quality such as teacher–child interactions and the characteristics of the pedagogical activities. However, it is important to note that the ERS scales are not generally thought to be accurate measures of other relevant dimensions of process quality such as individualized attention and positive emotional climate, and the evidence is mixed regarding their power to predict child development (Mashburn et al., 2008; Burchinal et al., 2000; Sylva et al., 2006).

We collected ECERS for 119 classrooms in all CDIs at follow-up, ITERS for 37 classrooms in all CDIs at follow-up, and FDCRS for 54 HCBs still running at follow-up. These instruments were not collected at baseline. The ECERS, ITERS and FDCRS capture the same constructs to the extent that is possible. However, it is clear that they measure specific features in each type of setting. No studies are available that compare the three scales. According to the test developers, scores from these tests are often treated as if they are comparable, particularly in the U.S. literature.

For example, Fuller, Kagan, Loeb, and Chang (2004) assess the quality of center-based and home-based child care for low-income children in four sites in the U.S. using the ERS scales. The authors find that home-based settings formally arranged for children in ways that resemble centers score higher than kith and kin settings in FDCRS. However, home-based settings − different from kith and kin − score lower than centers (ECERS). This result provides some evidence that our comparison is reasonable, as the home-based setting analyzed in this study resembles more the arrangements in centers than in kith and kin settings.

The ERS measures have been widely used in countries in the region similar to Colombia, such as Ecuador, Peru, Bolivia, Chile, and Brazil (Araujo et al., 2015; Berlinksy & Schady, 2015; Verdisco & Pérez Alfaro, 2010). In this study, ERS scores are positively correlated with the care provider’s years of experience and years of schooling, and negatively correlated with the classroom’s adult–child ratio.

### 5.5 Statistical analysis

The hypothesis that we tested was whether the offer to transfer from HCB to CDI had a positive or negative average impact on child development and nutrition. Treatment was randomly assigned given the cluster-randomized effectiveness trial design. We estimate intent-to-treat effects (*ITT*) by Ordinary Least Squares using the following specification:

(1) $$A_{i,h,t}=\lambda_{1}+\lambda_{2}ITT_{i,h}+\lambda_{3}A_{i,h,t-1}+\lambda_{4}X_{i,h,t-1}+\varepsilon_{i,h,t}$$

*ITT_i,h_* is a binary variable that equals 1 if child *i* from HCB *h* was randomly assigned to treatment, i.e., offered to transfer from an HCB to a center, and 0 otherwise. *A*_*i*,*h*,*t*_ is an outcome variable for child *i* from HCB *h* in period *t*, *A*_*i*,*h*,*t*−1_ is the same outcome for child *i* in HCB *h* but measured at baseline (or one in the same developmental domain if it was not measured at baseline), and *X*_*i*,*h*,*t*−1_ is a vector of baseline control variables that includes child’s gender, age and age squared in months, birth order, maternal years of schooling, indicators for whether the father and the mother are present, female-headed household, employment status of the head of household, household size, household wealth quintile, number of children in the household, the mean of the outcome variable in the HCB at baseline, years of schooling and working experience of the MC in the HCB at baseline, and city fixed effects. *ε*_*i*,*h*,*t*_ reflect unobserved factors that influence child outcomes.

*λ*_2_ captures the impact of *ITT* on the outcome, that is, the causal effect of having been offered the chance to move from HCB to CDI, regardless of whether individuals complied with the offer. To assess how the offer to transfer affected parental childcare choices, we also estimated Eq. (1) using as dependent variable the type of early education program used at follow-up.

We used two-tailed tests in our statistical analysis and cluster standard errors at the HCB level to account for the clustering of children by HCB at baseline. We also adjusted the *p*-values of estimated effects to correct for multiple hypotheses testing, using the step-down procedure proposed by Romano and Wolf (2005) − RW. In particular, the RW-adjusted *p*-values take into account the fact that within each developmental domain we tested multiple hypotheses by using several outcome measures.

We also estimated treatment-on-treated (*TOT*) effects. That is, *ITT* effects adjusted by the take-up rate, which take into account the fact that not all children took up the offer to transfer from HCB to CDI. We estimated *TOT* effects using an instrumental variable approach. In particular, we used random assignment (*ITT*) as an instrument for actual transfer from an HC to a CDI. In particular, we define a binary variable that equals 1 if child *i* from HCB *h* is enrolled in CDI at follow-up (i.e. registered in CDI rosters, not actual daily attendance), and 0 otherwise. Enrollment is directly obtained from CDI administrative records. Given that such a variable is (at least partly) determined by individuals’ choices that could affect children outcomes, we cannot identify the effect of switching from an HCB to a CDI with a standard regression. Using the *ITT* indicator as an instrument for actual transfer provides an unbiased estimate of the program’s effects among those who actually made the transition from HCB to CDI. The *ITT* indicator is a valid instrumental variable as it was randomly assigned, and it significantly explains CDI enrollment. A regression of enrollment in CDI on random assignment yields a statistically significant positive coefficient with an *F*-test of 9.96. Unfortunately, we do not have information about daily attendance.

We aggregate outcomes into a few developmental domains rather than using all developmental measures individually, in order to attain estimates that are more precise. To aggregate the outcomes, we conducted an exploratory factor analysis on all developmental outcomes available at follow-up, and constructed a dedicated measurement system. In particular, we included four ASQ subscales (communication, problem resolution, gross and fine motor), five WM-III subscales (general verbal ability, associative memory, executive function, mathematical reasoning and receptive language), seven ASQ:SE subscales (compliance, communication, adaptive functioning, autonomy, affect and interaction), two anthropometric measures (height and weight for age) and two health measures (anemia and parasites). We excluded from this exploratory analysis the arm-circumference because WHO standards are not available for children older than five years of age. The dedicated measurement system required that (i) factors selected had eigenvalues higher than one, (ii) items that heavily loaded in more than one factor were excluded, and (iii) items with factor loadings lower than 0.2 were also excluded.

This analysis yielded the following results. First, the exploratory analysis identified four main developmental factors: cognitive development by ASQ, cognitive development by WM, socio emotional development by ASQ:SE (excluding autonomy which did not load into any factor) and a nutrition/health factor which included height-for-age and weight-for age. Anemia and parasites did not load into any factor so these were excluded. Given that overweight and obesity are not very prevalent in this sample (see Table 1) we assumed that a higher nutrition factor was always better. We estimated Eq. (1) using these four factors as dependent variables. However, we also report impacts by individual developmental outcomes as a robustness check.

**Table 1:** Descriptive statistics at baseline.

	*ITT* = 0		*ITT* = 1	
	Mean	SD	Mean	SD
**Sociodemographic**				
Child's age in months	44.15	(10.32)	44.16	(10.6)
Child's gender (male)	0.51	(0.50)	0.51	(0.50)
Maternal yrs of schooling	10.08	(3.11)	9.90	(3.07)
Father present	0.62	(0.49)	0.64	(0.48)
Mother present	0.95	(0.22)	0.94	(0.24)
Female head of household	0.30	(0.46)	0.29	(0.45)
Household head working	0.88	(0.33)	0.86	(0.34)
Household size	5.19	(2.14)	5.23	(2.18)
0–5 children at home	1.52	(0.70)	1.54	(0.72)
Wealth quintile 1	0.18	(0.39)	0.21	(0.40)
Wealth quintile 5	0.20	(0.40)	0.19	(0.39)
No. of observations	700		2067	
**Outcomes**				
***Cognitive development*** a				
ASQ communication	−0.03	(1.00)	0.01	(1.00)
ASQ problems resolution	−0.03	(0.98)	0.01	(1.00)
ASQ gross motor	0.01	(1.01)	0.00	(0.99)
ASQ fine motor	0.01	(0.94)	0.00	(1.02)
No. of observations	700		2066	
***Socio-emotional development***^a^				
ASQ:SE total	0.04	(1.00)	−0.01	(1.00)
No. of observations	700		2066	
***Nutrition***				
Weight for age *Z*-score	−0.47	(0.93)	−0.48	(0.95)
Length for age *Z*-score	−1.02	(1.02)	−1.06	(1.00)
Weight for length *Z*-score	0.20	(0.92)	0.20	(0.92)
Arm circumference-for-age *Z*-score	0.10	(0.84)	0.08	(0.81)
Underweight	0.04	(0.20)	0.04	(0.21)
Wasting	0.00	(0.07)	0.01	(0.09)
Stunting	0.17	(0.37)	0.16	(0.37)
Overweight	0.02	(0.14)	0.03	(0.16)
Obesity	0.01	(0.11)	0.01	(0.07)
No. of observations	605		1.700	

Note: ***p < 0.01; **p < 0.05; *p < 0.1.

Cluster-robust standard errors.

a Internally (non-parametrically) age-standardized scores reported.

We also investigated the possibility of heterogeneous impacts by key baseline characteristics. In particular, we looked into differential effects by child’s gender and age, and potential duration of exposure to the program. The latter is proxied by the amount of rainfall in a given city during the time elapsed between baseline and center opening as construction delays occurred due to a climatic phenomen known as *La Niña*. Thus, while duration of exposure to the program might be endogenous (and we do not have data on actualy daily attendance), the amount of rainfall is plausibly exogenous to unobserved characteristics of children.

Finally, we also explored the extent to which these impacts might be explained by differences in the quality of both services during the transition. To this aim, we present differences in classroom quality between children served in CDIs and children served in HCBs at follow-up using the ERS scales.

## 6 Results

In this section, we present descriptive statistics on the sample, discuss the compliance with random assignment, and present estimates of the effects of the offer to transfer from HCB to CDI on child development and health. We also present the effects of the offer on the type of early education program children attended and the differences in quality of care provided to them at follow-up.

### 6.1 Descriptive statistics

Table 1 provides summary statistics at baseline by treatment group. We included sociodemographic characteristics of children, as well as developmental outcomes. Children were, on average, 44 months of age, with about 30% of the households being single-headed, and 1.5 children younger than five per household. Maternal average schooling was 10 years, and 38% of children did not live with their fathers. Average height-for-age and weight-for-age are 1.5 and 0.5 SD below the mean of the reference population, respectively. Nutritional status in our sample is worse than that of comparable children in Colombia’s national longitudinal survey (ELCA, 2013). In particular, stunting is about 17% in our sample compared to 13% for the lowest urban SES in Colombia and 10% nationwide.

We did not collect WM-III, haemoglobin or stool sample at baseline so these are not reported in Table 1. At follow-up, 28% of children were anemic and 53% had parasites. In sum, children in this sample had a high developmental risk. Table 1 also provides confirmation of baseline balance between study groups. Overall, there are no statistically significant differences between *ITT* groups at baseline, and the differences observed are not systematically in favor of one of the two groups. Baseline characteristics do not significantly predict *ITT*.

### 6.2 Compliance with random assignment to treatment

Close to 75% of MCs assigned to treatment transitioned to CDI, while 60% of MCs assigned to control did not transfer. By moving to centers, MCs were expected to change from working in their own home to working in the CDI, from being self-managing their work to having an on-site line manager, and from having flexibility in their daily activities to having specific guidelines about how their time should be allocated, including having a formal schedule and specific responsibilities that were closely monitored by center directors. On the other hand, the transfer might have had positive features such as being released from administrative duties, and cooking and cleaning responsibilities. In the absence of a wage change associated to the transfer from HCB to CDI, a fraction of MCs did not agree to the transfer, possibly because the disadvantages outweighed the advantages.

Looking at reasons for noncompliance, we found that 22% of non-complying MCs did not want to transfer, 11% were requested by parents not to transfer, 27% reported personal problems, 16% mentioned health-related issues, and 11% had not completed high-school. At follow-up, 89% of all teachers working in centers had previously worked as MCs. We also found that MCs who chose not to move to centers were, on average, older, had more work experience and were less educated than both, MCs who chose to transition to centers, and CDI teachers who had not worked as MCs. 22% of MCs that did not transition to CDI centers had completed a degree in higher education, while close to 55% of both, MCs in centers and new teachers, had.

In terms of children’s compliance with the random assignment, we observed significant non-compliance. Close to 22% of the children in HCBs that received an offer to transfer to a CDI were effectively enrolled in a CDI at follow-up, and 16% of children of HCBs that did not receive an offer to transfer were enrolled in a CDI at follow-up. This difference is statistically significant, implying that the random assignment did have a role, albeit limited, in determining CDI attendance. Looking at compliance by children age, we find that 39% of children younger than three assigned to the treatment were enrolled in CDI at follow-up while only 14% of children older than three were. In Table 2, we study the extent of the cross-over in compliance by MCs and children attending their HCBs. In particular, we show the degree of compliance of MCs by study group, and add the fraction of compliers and non-compliers who transferred (not transferred) to CDI with less than 50% of their children at baseline and with more than 50% of their children at baseline.

**Table 2:** Compliance of MCs with random assignment and attendance decisions at follow-up of children served by them at baseline.

	Transferred to CDI	Transferred to CDI		Did not transfer	Did not transfer to CDI	
		≤50% children followed	>50% children followed		≤50% children followed	>50% children followed
ITT = 1	75.3%	80.4%	19.6%	24.7%	96.4%	3.6%
ITT = 0	39.2%	86.2%	13.8%	60.8%	82.2%	17.8%

Table 2 shows that 75% of MCs assigned to treatment transferred from HCB to CDI. Out of these, 80% transferred with less than 50% of their own baseline children (still age-eligible at follow-up), and 20% transferred with more than 50% of their baseline children. Similarly, 61% of MCs assigned to the control group did not transfer from HCB to CDI. Out of these, 82% were serving less than half their (still age-eligible) baseline children at follow-up, and 18% were still serving more than 50% of their baseline children at follow-up. In sum, most of non-compliance decisions occurred at the level of the household and not on the part of MCs. Parents significantly deviated from the decisions of MCs, whether they had been assigned to treatment or control.

In Table 3, we show characteristics of children by study group and compliance status. Children, who did not transfer to CDI, regardless of their random assignment, were significantly older and were less likely to reside in a female-headed household than those who transferred to CDI. Non-compliers in the treatment group were more likely to have mothers with lower education and reside in larger households than compliers. They also exhibited higher communication ASQ scores and higher ASQ gross motor scores (at 90% confidence), and fewer behavioral problems than children in the treatment group who complied with random assignment. Non-compliers in the control group were less likely to have younger siblings, exhibited lower problem resolution ASQ scores and lower height-for-age than children who stayed in HCB. There are no differences between compliers and non-compliers in household wealth in either study group.

**Table 3:** Characteristics of children at baseline by study group and compliance status.

	ITT = 0					ITT = 1					
	Compliers (C)		Non-compliers (NC)		Signif. diff	Compliers (C)		Non-compliers (NC)		Signif. diff	Signif double diff b
	Mean	SD	Mean	SD		Mean	SD	Mean	SD		
**Sociodemographic**											
Child's age in months	45.23	(10.13)	37.98	(8.96)	***	37.41	(9.35)	46.00	(10.08)	***	***
Child's gender (male)	0.51	(0.50)	0.53	(0.50)		0.52	(0.50)	0.50	(0.50)		
Maternal yrs of schooling	10.20	(3.11)	9.74	(3.02)		10.36	(2.93)	9.82	(3.10)	***	
Father present	0.63	(0.48)	0.61	(0.49)		0.63	(0.48)	0.67	(0.47)		
Female head of household	0.27	(0.44)	0.41	(0.49)	**	0.33	(0.47)	0.27	(0.44)	**	***
Household size	5.26	(2.13)	4.92	(2.01)		4.95	(2.10)	5.34	(2.17)	***	***
0–5 children at home	1.54	(0.70)	1.38	(0.63)	**	1.50	(0.77)	1.55	(0.72)		**
Wealth quintile 1	0.19	(0.39)	0.16	(0.37)		0.18	(0.39)	0.20	(0.40)		
Wealth quintile 5	0.20	(0.40)	0.20	(0.40)		0.20	(0.40)	0.19	(0.40)		
No. of observations	523		99			408		1,425			
**Cognitive development**^a^											
ASQ communication	−0.03	(0.98)	−0.06	(1.10)		−0.10	(1.06)	0.05	(0.97)	**	
ASQ problems resolution	0.01	(0.97)	−0.23	(0.99)	**	−0.06	(1.04)	0.03	(0.99)		**
ASQ gross motor	0.02	(1.00)	−0.05	(1.03)		−0.09	(0.96)	0.01	(1.00)	*	
ASQ fine motor	0.04	(0.93)	−0.14	(0.99)		−0.04	(1.07)	0.00	(1.00)		*
No. of observations	523		99			408		1,424			
**Socio-emotional dev.** a											
ASQ:SE total	0.07	(1.03)	−0.07	(0.90)		0.09	(0.99)	−0.04	(1.01)	**	
No. of observations	523		99			408		1.424			
**Nutrition**											
Weight for age *Z*-score	−0.46	(0.89)	−0.55	(0.98)		−0.47	(0.92)	−0.47	(0.97)		
Height for age *Z*-score	−0.99	(0.98)	−1.20	(1.14)	*	−1.07	(0.97)	−1.06	(1.01)		*
No. of observations	482		94			386		1,251			

Note: ***p < 0.01; **p < 0.05; *p < 0.1. Cluster-robust standard errors and *t*-tests adjusted for HCB clustering at baseline.

a Non-parametrically age-standardized ASQ scores.

b Double difference computed as: (compliers' mean − non-compliers' mean | ITT=1) − (compliers' mean − non-compliers' mean | ITT=0).

More than half of non-complying children in the treatment group did not transfer to CDIs because they were too close to be reaching the maximum age for eligibility, as reported by parents. Also, 17% had transferred to pre-primary education in public schools by the time centers opened, and 16% reported that the center was too far. Albeit the fact that the transfer from HCB to CDI required a 1-km proximity criterion, this distance was in most cases larger that the distance between households and HCBs which is, on average, less than a 10-min walk (Bernal et al., 2009).

In sum, a key determinant for the transition from HCB to CDI was the child’s age with children close to the age-eligibility limit, being less likely to transfer. Apart from that, non-compliers in the treatment group seem to reside in households that were more vulnerable by size and maternal education (not by wealth) but had higher cognitive scores by ASQ. These differences are controlled for in the estimation of *TOT* effects, by virtue of the difference-in-difference specification in Eq. (1) and the instrumental variable approach proposed.

### 6.3 Results on the choice of early education service

In Table 4, we present the *ITT* impacts of the offer to transfer from HCB to CDI on the ECE program that the child was enrolled in at follow-up. The dependent variable was defined as a categorical variable indicating if child *i* was enrolled in CDI, HCB, or other early education program at follow-up, or was not enrolled in any early education program, based on parental reports. We estimated it by using a multinomial regression model.

**Table 4:** Intent-to-treat effect on type of childcare enrollment.

Enrollment by type of program at follow-up	*ITT* estimate
CDI	0.132***
	(0.04)
HCB	−0.114***
	(0.04)
Other early education service	−0.027
	(0.04)
Not enrolled	0.010
	(0.02)
No. of observations	2455
Wald chi^2^ (105)	1.258
Prob > chi^2^	0.0000
Pseudo *R*^2^	0.3345

Note: ***p < 0.01; **p < 0.05; *p < 0.1.

Model estimated by multinomial logit. Marginal effects reported. Cluster robust standard errors in parentheses. The specification includes the same covariates as in Eq. (1).

The results indicate that being offered the chance to transfer from HCB to CDI did significantly increase CDI enrollment at follow-up by 13 percentage points and decreased HCB enrollment at follow-up by 11 percentage points. The offer did not have any statistically significant effects on attendance to other programs or not using childcare. These results indicate that while the study might be underpowered due to noncompliance, the offer to transfer from one service to another did have the expected effects on the choice of childcare program.

### 6.4 Results on child development and health

Table 5 reports the *ITT* impacts on the four developmental outcomes described earlier: cognitive development by ASQ, cognitive development by WM, socio-emotional development, and nutrition (left panel), as well as *TOT* estimates on these four outcomes (right panel). The results indicate that the *offer* to transfer from HCB to CDI had a negative and statistically significant effect on cognitive development by ASQ of 0.11 standard deviations (SD) at 95% confidence level. At the same time, the offer had a positive effect on nutrition of 0.05 SD at 95% confidence level. Similar results can be observed for *TOT* effects reported in the right panel of Table 1. There is a negative effect of the treatment-on-the-treated on cognitive development by ASQ of 1.5 SD but with a very large confidence interval. In particular, we cannot reject the null hypothesis of null effects on ASQ at 95% confidence level. We also report a positive effect of 0.6 SD on nutrition at 90% confidence. The effect on the WM cognitive factor exhibits a negative sign but is not statistically significant. Similarly, the effect of the offer and the effect of the *TOT* on socioemotional development is positive (because it decreases behavioral problems) but is not statistically significant.

**Table 5:** Intent-to-treat and treatment-on-treated effects on children's developmental factors.

Outcomes		*ITT* estimates^a^			*TOT* estimates^b^		
	*N*	Effect	Std Error	*p*-Value	Effect	Std error	*p*-Value
ASQ cognitive factor	2435	−0.11	(0.05)	0.04**	−1.52	(0.89)	0.09*
WM cognitive factor	1905	−0.01	(0.05)	0.85	−0.13	(0.72)	0.85
ASQ socio-emotional factor	2429	−0.02	(0.05)	0.67	−0.29	(0.69)	0.67
Nutrition/health factor	2159	0.05	(0.02)	0.03**	0.58	(0.33)	0.08*

Note: ***p < 0.01; **p < 0.05; *p < 0.1. Cluster robust standard errors in parentheses.

The covariates included are listed below Eq. (1).

a OLS estimates.

b Two-stage least squares estimator; uses random assignment as an instrument to predict program compliance.

In Appendix A, we report the same results for individual developmental measures instead of factors. As can be observed, we report only a statistically significant increase in the probability of wasting at 95% confidence level. All other *ITT* and *TOT* effects are statistically insignificant. The direction of the effects coincides for most part with those presented in Table 3. However, due to the low power of the study and the proliferation of measures, which required adjustment for multiple hypotheses testing, these effects are imprecisely estimated.

In sum, the results indicate positive program effects on nutrition and suggest negative effects on cognitive development. The effect on nutrition is small representing about 5% of a standard deviation. This result might be due to the presence of a professional nutritionist in CDIs who is in charge of overseeing all processes related to food provision in centers (purchases, reviews of menus, revision of portions), as well as anthropometric follow-up and monitoring of children at nutritional risk, and involvement with families to improve nutrition and health habits. While both programs provided the same fraction of daily nutritional requirements, it is possible that the content was better in CDI than in HCB, and that there was better monitoring of the process of ensuring food quality and overseeing feeding practices in the centers. Unfortunately, we do not have data on these processes to corroborate these hypotheses.

On the other hand, the results suggest negative effects on cognitive development. We are cautious in interpreting this finding as the ASQ is generally regarded as a screener test and not considered a gold-standard measure of child development. The cognitive factors included dimensions such as language, problem resolution, mathematical reasoning and memory. These abilities are probably more closely associated with teacher–child interaction and the learning environment in the classroom than nutrition is. The negative or null effects on cognition might be indicative of the fact that the transition from CDI to HCB did not explicitly change process quality features such as teacher pedagogical and classroom organization skills, a developmentally focused curriculum, and a language-rich and responsive environment (Yoshikawa et al., 2016). In other words, the mere transfer to a different physical space, the age-grouping of children and the presence of more qualified staff was not enough to guarantee significant changes in the learning environment at CDIs with respect to HCBs. We explored this hypothesis further by analyzing data on classroom quality (see below).

We also looked into the effects for specific groups of children. In particular, we analyzed heterogeneous effects on developmental factors by child’s age and sex, and the amount of rainfall in the municipality of residence. These results are not shown but are available upon request. We did not find any differences by child’s age (neither by *ITT* nor by *TOT*). We observed a negative *ITT* effect on cognitive development by ASQ that was higher for boys (−0.13 SD) than for girls (−0.08 SD) but the difference was not statistically significant. No statistically significant differences were observed by *TOT*.

Children in this study could have been exposed to the intervention for different lengths of time mostly due to delays in the construction of the centers associated with the *La Niña* climatic phenomenon. We used the amount of rainfall in the child’s town of residence between baseline and the opening of the center as a proxy for the duration of exposure to the intervention. We did not observe any differences in program impacts depending on how long children could have been exposed to the intervention. This is important because, being a new initiative, CDIs might have required some time to optimize procedures. It is possible that the transition period required to achieve an optimized model was longer than the duration of the evaluation study.

### 6.5 Differences in childcare quality by program

In Table 6, we present differences in classroom quality as measured in 113 CDI classrooms of children older than two years of age by ECERS, 36 CDI classrooms of infants and toddlers by ITERS and 54 HCBs by FDCRS, at follow-up. In panel I, we show total scores computed by averaging over all items (Cronbach’s *α* = 0.76 for ECERS, 0.52 for ITERS and 0.81 for FDCRS), as well as total scores split in two categories: the space & furnishings subscale (Cronbach’s *α* =0.40 for FDCRS but undetermined for ECERS and ITERS due to a negative covariance among items) and all the other subscales excluding space & furnishings (Cronbach’s *α* =0.76 for ECERS, 0.52 for ITERS and 0.79 for FDCRS). The Space & Furnishings subscale includes items that characterize the indoor space, the furniture, the room arrangement, the space and equipment for motor gross play, and the space available for child privacy (i.e., items related to the infrastructure). The rest of the subscales are more closely related to what children actually experience in the program. In panel II, we show scores for all the seven subscales available in the ERS scales. In particular, we included the personal care routines subscale (Cronbach’s *α* lower than 0.2 in all cases), language and reasoning (Cronbach’s *α* between 0.4 and 0.55), activities (Cronbach’s *α* is 0.33 for ECERS/ITERS and 0.75 for FDCRS), interaction (Cronbach’s *α* =0.74 for ECERS, 0.44 for ITERS and 0.24 for FDCRS), program structure (Cronbach’s *α* lower than 0.4 for ECERS/ITERS − the scale is not available in FDCRS), and parents and staff (Cronbach’s *α* =0.63 for ECERS, 0.46 for ITERS and 0.53 for FDCRS).

**Table 6:** Differences in childcare quality by childcare modality.

Quality scores	CDI			HCBs		
	ECERS	ITERS	TOTAL (A)	FDCRS (B)	Diff CDI vs HCB (A) - (B)	Diff *p*-value
	*N* = 113	*N* = 36	*N* = 149	*N* = 54		
I. Total scale score^a^	1.72	1.85	1.75	2.19	−0.44	0.000***
	(0.56)	(0.26)	(0.58)	(0.41)	(0.07)	
Space & furnishings subscale	1.64	1.89	1.70	1.52	0.18	0.011**
	(0.53)	(0.29)	(0.51)	(0.45)	(0.07)	
Score excl. space and furnishings	1.74	1.84	1.76	2.32	−0.56	0.000***
	(0.59)	(0.28)	(0.61)	(0.46)	(0.08)	
II. Subscales^a^						
Space and furnishings	1.64	1.89	1.70	1.52	0.18	0.011**
	(0.53)	(0.29)	(0.51)	(0.45)	(0.07)	
Personal care routines	1.38	1.21	1.34	1.67	−0.33	0.000***
	(0.56)	(0.20)	(0.52)	(0.36)	(0.06)	
Language and reasoning	1.70	1.85	1.74	2.17	−0.43	0.000***
	(0.82)	(0.63)	(0.89)	(0.70)	(0.12)	
Activities	1.49	1.47	1.48	2.27	−0.79	0.000***
	(0.51)	(0.35)	(0.52)	(0.62)	(0.09)	
Interaction	2.07	2.17	2.09	2.11	−0.01	0.916
	(1.05)	(0.71)	(1.13)	(0.67)	(0.13)	
Program structure	1.59	2.07	1.70	NA	NA	NA
	(0.92)	(0.60)	(1.00)			
Parents and staff	2.20	2.27	2.21	3.39	−1.18	0.000***
	(1.63)	(0.94)	(1.80)	(1.04)	(0.20)	

Note: ***p < 0.01; **p < 0.05; *p < 0.1. Standard deviations of means and standard errors of differences in parentheses.

The *t*-test for the differences between CDI and HCB adjusted for CDI clusters.

Standard deviations of means in columns (2) and (3) adjusted for CDI clusters.

ECERS: Early Childhood Environmental Rating Scale (Harms, Clifford, and Cryer, 1998).

ITERS: Infants and Toddlers Environmental Rating Scale (Harms, Cryer, and Clifford, 2003).

FDCRS: Family Day Care Rating Scale (Harms and Clifford, 1989).

For total scores or subscales, the score was calculated as an average rating of all items.

As it is uncommon to have this type of data available in evaluations of large scale programs, we chose to present these results as suggestive evidence that can explain the program impacts reported above; however, we acknowledge the potential limitations of the ERS quality indicators discussed earlier in the Methods section.

The results indicate that both, HCBs and CDIs, had inadequate quality levels by this measure. The results are similar to those reported in Bernal and Fernandez (2013) using FDCRS scores in a sample of 400 HCBs nationwide. Using similar instruments, a variety of ECE programs in the Latin-American region have been shown to exhibit similar low levels of quality (Araujo, López Boo, Novella, Schodt, & Tomé, 2015; Berlinski & Schady, 2015; Verdisco & Pérez Alfaro, 2010). Similarly, Maldonado-Carreño and Votruba-Drzal (2014) show that the quality of private ECE for socioeconomically vulnerable populations in Colombia is not significantly better than that found for the public ECE programs under study.

Overall and perhaps surprisingly, quality scores were significantly higher in HCB (total score = 2.19) than in CDI classrooms (total score = 1.75). The computation of the *t*-test for the difference between CDI and HCB adjusted for CDI clusters. When we analyzed the Space & Furnishings subscale separately from the rest, we found that structural quality (as measured by these items) is significantly higher in CDI than in HCB, as expected. The difference is statistically significant at 95% confidence level. However, the average of all items excluding the Space & Furnishing subscale exhibited significantly higher quality in HCB than in CDI. The difference of about 0.56 scale-points (or 1.3 SD of FDCRS) is large. Fuller, Kagan, Loeb, and Chang (2004) report higher scores for center-based child care with respect to home-based settings serving low-income children, such as the one analyzed in this study, in four sites in the U.S. using the same ERS comparison. These results are consistent with other studies using these or other instruments especially in low-income settings (NICHD, 1997; Clarke-Stewart, 1999). The fact that we find the opposite, might suggest that quality in new centers was not fully consolidated by the end of this study. When looking into individual subscales, we observed statistically significant differences in favor of HCB in all but: space & furnishings and interactions. Other than that, HCBs scored better in personal care routines, language and reasoning, activities, and parents and staff.

One important issue to bear in mind when interpreting these results is the fact that CDIs were newly developed centers at the time of the evaluation study. As with any new program, the optimization of procedures can take time. It is possible that these results reflect a transition period and that quality might have improved as the program established. In fact, when we compare centers with less than 12 months of operation with centers with more than 12 months of operation (the median), we observe that the latter exhibit better quality scores than the former. The difference is close to half a standard deviation and it is statistically significant for space & furnishings, language and reasoning, activities, and program structure. While this consideration grants some caution in the interpretation of our results, as CDI quality might have been still improving at the time of the follow-up, we notice that it is still lower (except for space and furnishings) than in HCBs in both cases.

These results suggest that during this transition period, the differences in quality, and particularly, differences in quality aspects different from infrastructure, might have had a role in explaining the negative and null impacts on children’s cognitive and socio emotional development, respectively. However, the evolution of quality in centers was headed in the right direction.

## 7 Discussion and conclusion

To evaluate the impacts of the offer to transfer from small community nurseries to large centers on childcare quality and child development and nutrition, we randomly assigned HCBs to receive an offer to transfer to CDIs in 14 cities in Colombia. Despite of imperfect compliance with the random assignment, the results suggest negative effects of both, the offer to transfer from HCB to CDI and the actual transfer to CDIs, on children’s cognitive development, as well as positive *ITT* and *TOT* program impacts on nutrition. No significant effects were found on socioemotional development.

The significant improvement in nutrition might be associated with the presence of a professional nutritionist in CDIs, who is in charge of all processes related to food provided to children in centers. In contrast, the abilities considered in the cognitive factor such as language, mathematical reasoning and problem resolution are plausibly more closely associated with teacher–child interactions and the learning environment in the classroom than nutritional status. As changes associated with the transfer from HCB to CDI related more explicitly to physical infrastructure, age-specific classrooms, and more qualified staff, it was not clear that process quality (e.g., teacher pedagogical strategies and responsive learning environments) improved. Comparisons of quality in CDIs and HCBs by ERS scales provided some evidence in favor of this hypothesis, as these showed that infrastructure was, in fact, better in CDIs than in HCBs, while all other aspects more closely related to what children actually experience in the program exhibited higher scores in HCBs than in CDIs. It is important to mention that CDIs did have comprehensive operational and technical guidelines (or referents). The results point to the fact that those guidelines did not provide pedagogical or operational directives of enough specificity to drive process quality improvements as intended.

In understanding these results, it is important to keep in mind a few facts. First, the ERS scale might not be the best way to measure relevant dimensions of process quality such as positive interactions, individualized attention and positive emotional climate. Second, as CDIs were newly developed centers at the time of the evaluation study and new programs might take time to achieve optimal procedures, we might be measuring both, program effects and program quality during the transition period only. In fact, centers that had been operating for a longer time exhibited significantly higher scores than centers that had been operating for shorter time, implying that quality was improving in the right direction over time. However, both were still lower in CDI than in HCBs (with the exception of spaces & furnishings) by the end of the study. In this sense, it is crucial to keep monitoring the quality of CDIs over time, to explore if process quality improves after the new program has had some time to get established. Third, we found that MCs who chose to move to centers had, on average, less work experience than MCs who did not transition to centers. As time of professional experience could be a significant mediator of process quality, this could be an important factor in explaining the results found in this study. Thus, it is important to study further how to support new staff with low work experience, particularly in terms of the implementation of key features of process quality.

Fourth, this study did not address the predictive power of ERS scales on child development and the literature is inconclusive in this sense. It is clear, then, that further research is needed to assess more systematically the effects of different elements of ECE quality on child development. Finally, one must keep in mind that children in our study sample shared CDI classrooms and HCBs with children not included in the study (e.g. HCBs prioritized for the transfer because of extreme vulnerability). In fact, non-study children in CDIs at follow-up came from poorer households and scored significantly lower in cognitive ASQ than study children. There were no significant differences in terms of socio-emotional development nor anything related to health. It is possible that, in the presence of peer effects, the presence of children from more disadvantaged background could partly explain the results presented here. However, it is unlikely that this would be the main reason, especially if the difference is not due to misbehavior in large CDI classrooms (as captured by ASQ socio-emotional).

The main limitation of this study is that compliance with the random assignment to transfer from HCB to CDI was imperfect. This issue does not invalidate the experimental design, as attendance to a CDI is significantly more likely for the treated than the control group, so that, as we discuss above, the random assignment can be used as an instrumental variable to identify (at least for the compliers) the impact of the transfer. However, the low level of compliance might imply that the study could be underpowered to detect statistically significant program effects. In addition, the requirement to adjust *p*-values to take into account of multiple hypotheses testing exacerbates the problem of insufficient power, especially given our rich list of developmental outcomes.

Although there is no perfect solution for this issue, we tried to alleviate this concern by aggregating outcomes into a few developmental domains rather than using all developmental measures individually in order to improve precision, and by discussing in detail the differences between groups of children by their compliance.

It is also important to keep in mind that the imperfect compliance of the study also somewhat limits the external validity of the results presented. In particular, the TOT estimates reported are identified out of those households whose decision to switch from a HCB to CDI was affected by the offer, that is, the compliers. The effect on the non-compliers can be, in principle, different, and our experiment has no way to identify it. We do present some evidence on the features that characterize the compliers. While differences in terms of children age are not particularly worrying (the parents of a child close to switch to school might want to avoid disruption and changes), those in terms of parental background and socio-economic status could be of concern as the impacts on non-complier groups could be substantially different from what we estimate.

The issue of low compliance to CDI enrollment is an important finding in and of itself. It poses the question about how to more systematically assess these barriers to program access, understand how these affect attendance and adherence, and design strategies in order to overcome those barriers. For example, 16% of the non-compliance of treated children reported that the CDI was too far, even though CDI centers were, at most, within one kilometer from HCBs. It is important that policy design take distance and other convenience features into consideration as these could potentially impact not only enrollment but also attendance to the program.

Another limitation of this study, is the possibility that developmental outcomes might have been collected with measurement error, particularly those reported by parents (such as health, ASQ and ASQ:SE), and that these specific measures lack enough sensitivity due to the fact that these are commonly used as a screener and not as a gold-standard measure of child development. However, these measures have been complemented with more reliable direct measures such as the Woodcock-Muñoz and anthropometric measures. Third, we did not collect daily attendance data, which prevents us from studying more rigorously the effects of actual duration of exposure to the program. We have tried to overcome this issue by presenting heterogeneous effects by rainfall which proxies for delays in center opening.

The results of this study show that although modernization of early childhood services is desirable, it is critical that quality in some specific dimensions, related to processes, is specifically targeted and monitored during the transition. The findings in this study and the comparative costs of the new centers and the existing HCBs suggest that the strategy should be thought through carefully. Alternatives might have to be tested, including improvements to HCBs and training of MCs (Bernal, 2015). Different pedagogical models should also be assessed and adult–child ratios carefully considered. However, these results do not necessarily imply that center-based childcare cannot have positive effects, if implemented with the characteristics required to guarantee key elements of structural and process quality (Britto et al., 2011) and if programs are adequately supported during the transition in order to optimize procedures and ensure quality.

Some examples include the results presented by Nores, Bernal, and Barnett (2016) who assess a high-quality childcare center-based service in Colombia. The aeioTu program is characterized by high quality components such as high qualifications requirements for staff, pre- and in-service training, strong support staff and services, child monitoring and information system, family and community participation, and the use of a structured developmentally oriented curriculum. The authors reported positive effects on language and cognitive after only 8 months of program attendance. Similarly, Attanasio, Baker-Henningham, Bernal, Meghir, and Rubio-Codina (2017) reported positive effects on children’s cognition and language as a result of the implementation of a structured early stimulation curriculum in addition to training and coaching of paraprofessional personnel in a parenting program in rural areas in Colombia. Both are public programs targeted to poor households.

Thus, it is important that in implementing strategies to improve child care services for vulnerable children at scale, key factors such as the provision of teacher pre-service and in-service training, children assessment and monitoring, and strong curricular background are kept as a priority.

## Funding and role of the funding source

This study was funded by the Colombian Family Welfare Agency (*Instituto Colombiano de Bienestar Familiar,* ICBF) through interinstitutional agreements number 059-2010, 309-2011, 483-2011 and 2424-2012. The funding source supported the implementation of this study as the programs under evaluation are managed and funded by this institution. However, the institution did not participate in the study design; in the collection, analysis or interpretation of data; in the writing of the report; or in the decision to submit the article for publication. Professor Attanasio and Professor Vera-Hernández also received funding for this project from the European Research Council (ERC) under the European Union's Horizon 2020 research and innovation programme (grant agreement No 695300-HKADeC-ERC-2015-AdG).

## Figures and Tables

**Table:** 

Outcomes		*ITT* estimates^a^			*TOT* estimates^b^		
	*N*	Effect	Std error	RW *p*-value^c^	Effect	Std error	RW *p*-value^c^
***Cognitive development***							
ASQ communication	2448	−0.06	(0.05)	0.77	−0.86	(0.74)	0.77
ASQ problems resolution	2436	−0.06	(0.05)	0.77	−0.82	(0.69)	0.77
ASQ gross motor	2446	−0.11	(0.05)	0.16	−1.61	(0.86)	0.43
ASQ fine motor	2446	−0.10	(0.05)	0.44	−1.35	(0.86)	0.59
WM general verbal ability	2277	−0.22	(0.64)	0.99	−3.04	(8.78)	0.99
WM associative memory	2039	−0.19	(0.89)	0.99	−2.87	(13.09)	0.99
WM attention	2095	0.15	(0.61)	0.99	2.30	(9.55)	0.99
WM mathematical reasoning	2250	−0.69	(0.74)	0.85	−9.76	(10.74)	0.83
WM receptive language	2270	0.18	(0.55)	0.99	2.51	(7.49)	0.99
***Socio-emotional development***							
ASQ:SE total	2429	−0.03	(0.05)	NA	−0.38	(0.71)	NA
***Nutrition***							
Weight for age *z*-score	2169	0.03	(0.02)	0.55	0.36	(0.31)	0.62
Length for age *z*-score	2162	0.06	(0.02)	0.19	0.70	(0.37)	0.42
Weight for length *z*-score	1141	−0.09	(0.04)	0.19	−0.64	(0.35)	0.42
Arm circumference-for-age *z*-score	1156	−0.03	(0.05)	0.78	−0.23	(0.36)	0.80
Underweight	2169	0.01	(0.01)	0.42	0.12	(0.08)	0.53
Wasting	1141	0.01	(0.00)	0.03**	0.09	(0.04)	0.33
Stunting	2162	0.02	(0.01)	0.42	0.20	(0.13)	0.53
Overweight	2066	0.00	(0.01)	0.88	0.02	(0.13)	0.88
Obesity	2066	0.01	(0.01)	0.42	0.12	(0.08)	0.53
***Health***							
Anemic	2113	−0.01	(0.02)	0.85	−0.14	(0.28)	0.87
Parasites	2143	0.01	(0.03)	0.85	0.10	(0.33)	0.87

## Appendix A Intent-to-treat and treatment-on-treated effects on children’ outcomes

Note: ****p* < 0.01; ***p* < 0.05; **p* < 0.1. Cluster robust standard errors in parentheses.

The covariates included are listed below Eq. (1).

Total scores are not included in the RW procedure (hence it is shown as NA).

^a^OLS estimates.

^b^Two-stage least squares estimator; uses random assignment as an instrument to predict program compliance.

^c^The *p*-value obtained from the RW procedure for multiple hypotheses testing, within a developmental domain.

## Footnotes

2 By Colombia regulation, employers have the right to change employment conditions during the course of a valid contract, but if the changes are detrimental for the worker’s well-being then the worker is not obliged to comply with the request to change. Judges assess the possibility of negative effects on workers’ well-being. ICBF did not want to be exposed to potential lawsuits that could be justified on the grounds of significant changes resulting from the transfer from HCBs to CDIs.

3 The inter-rater reliability is a test-retest measure to provide evidence of the consistency of a test, and thus, the quality and reliability of the training provided to assessors. In this case, it measures the correlation between measurements by different assessors on the same subject.
